# The performance of the EQ-5D-Y-5L compared to the EQ-5D-Y-3L in children and adolescents with cerebral palsy (CP)

**DOI:** 10.1016/j.dialog.2022.100032

**Published:** 2022-08-01

**Authors:** Janine Verstraete, Des Scott

**Affiliations:** a Department of Paediatrics and Child Health, Division of Pulmonology, Cape Town, South Africa; b Faculty of Health and Rehabilitation Sciences, Division of Physiotherapy, Cape Town, South Africa

**Keywords:** Cerebral palsy, Children, Adolescents, Youth, Health related Quality of life, EQ-5D-Y, Three level, Five level

## Abstract

**Objectives:**

The aim of this study is to compare the performance and validity of the EQ-5D-Y-3L (Y-3L) and EQ-5D-Y-5L (Y-5L) in South African children and adolescents with cerebral palsy (CP).

**Methods:**

Children/adolescents with CP and those from the general population completed the Y-5L, Y-3L, and PedsQL. Physiotherapists at the school classified participants’ functional ability on the Gross Motor Functioning Classification System (GMFCS).

**Results:**

Fifty-one children/adolescents completed the measures. The ceiling effect had a 44% relative reduction for *Mobility* and floor effects decreased across all dimensions except for *Looking After Myself* when moving from the Y-3L to Y-5L. Informativity of dimensions improved on average by 0.27 on the Y-5L with similar evenness. There was a range of 6-16% inconsistent responses when moving from the Y-3L to the Y-5L. Convergent validity was strong on paired Y-3L and Y-5L dimensions: Kendall’s Tau B (range 0.53 – 0.85) and Gamma (range 0.79 – 0.99). There was significant moderate association between Y-3L and Y-5L with similar items on the PedsQL. The physical dimensions of *Mobility, Looking After Myself* and *Usual Activities* were significantly associated with GMFCS with those having less independent mobility reporting more severe problems on dimension scores.

**Conclusion:**

The Y-5L showed a notable reduction in ceiling and floor effects, improved discriminatory power, higher criterion validity with the GMFCS and similar concurrent validity with the PedsQL as the Y-3L. It is recommended that the Y-5L is further tested for reliability and responsiveness in this population group so that its utility for detecting change in clinical trials or as a routine outcome measure can be determined.

## 1 Background

Cerebral Palsy (CP) is a lifelong neuromotor disorder following non-progressive damage to the developing brain occurring prenatally or in in early childhood [1]. The global prevalence of CP is approximately 2.0 per 1000 live births [2], whereas in Africa the prevalence has been reported as up to five times higher [3]. CP is heterogenous with varying impairments and activity limitations based on the aetiology and the affected area and function of the brain [1]. The clinical classification of CP is based on the predominant motor abnormalities and the topographic distribution of symptoms but does not delineate functional abilities thus there will be a wide range of abilities within each group and across groups. As such the Gross Motor Functioning Classification System (GMFCS) is used to classify the level of motor functioning and gives a description of the child’s motor function and an indication of assisted technology the child might need in the future [4]. Apart from affecting physical performance, CP further impacts social and psychological functioning which can affect an individual’s quality of life [1,5].

Measurement of Health Related Quality of Life (HRQoL) is thus important in individuals with CP and can assist in prioritising treatment on an individual clinical level, studies of routine health care [6], clinical trials and with medical decision making [7]. Children who have the cognitive ability to self-report should be encouraged to do so on valid and reliable Patient Reported Outcome Measures (PROMs) [8]. The EQ-5D-Y, Child Utility Index – 9 Dimension (CHU-9D), Health Utility Index (HUI) and Assessment Quality of Life (AQoL) are all preference based PROMs which have been tested in children and adolescent with CP and are of particular interest as they are able to support medical decision making [7,9,10]. The EQ-5D-Y and CHU-9D are however the only measures that were developed or adapted for use in children aged between 8-15 years.

The levels of report on the EQ-5D-Y has recently been expanded from three levels on the EQ-5D-Y-3L (Y-3L) to five levels on the EQ-5D-Y-5L (Y-5L) [11]. The generic instrument measures health status for ‘Today’ across five dimensions *Mobility, Looking After Myself, Usual Activities, Pain or Discomfort* and *Worried, Sad or Unhappy* and a general rating of health on a Visual Analogue Scale (VAS) of 0-100 [12]. The increase in levels has shown a decrease in ceiling effect and improved discrimination in patient groups with acute and chronic illness [13], those receiving orthopaedic management [14] and idiopathic scoliosis [15,16]. The number of health states has increased from 243 (3^5^) on the Y-3L to 3125 (5^5^) on the Y-5L further improving its ability to detect change over time in patient groups [14,16]. This article presents a head-to-head comparison of the performance of the Y-3L and the expanded five level version, Y-5L in children and adolescents aged 8-15 years living with CP. The aim of this study was to investigate the feasibility, redistribution and discriminatory power of dimension responses, convergent validity, and criterion validity (concurrent and predictive validity) of the Y-3L and the Y-5L.

## 2 Methods

### 2.1 Study design and participants

An observational, cross-sectional study was conducted in children/adolescents with CP. Children/adolescents, aged 8-15 years, were recruited from four English-medium schools for learners with special education needs in the Western Cape, South Africa. These schools have specialised education and rehabilitation services for learners with normal intellect but who may require additional assistance or services due to a physical or learning disability. Physiotherapists at the participating schools identified learners with a diagnosis of CP for recruitment. Only those who were diagnosed with CP by a medical professional and who returned a signed informed consent and assent were included in the study. Those with an acquired gross motor disability due to other neurological insults such as tuberculosis meningitis, traumatic brain injury etc were excluded.

### 2.2 Instruments

#### 2.2.1 EQ-5D-Y

The official Y-3L English version for South Africa was used in this study. The experimental Y-5L English version for the United Kingdom was tested for equivalence in English for South Africa by the EuroQol group before it was used in this study. This Y-5L version was further tested for interpretation of severity qualifiers with the rank order task as described by Derrett et al (2021). The EQ-5D-Y consists of five dimensions namely *Mobility* (walking about), *Looking After Myself* (washing and dressing), *Usual Activities* (going to school, hobbies, sports, playing, doing things with family or friends), *Pain or Discomfort* and *Worried, Sad or Unhappy*. The original youth version, Y-3L, describes health on three levels (no problems, some problems and a lot of problems) [12,17] whereas the newly expanded version, Y-5L, describes health on five levels [no/not, a little bit, some/quiet, a lot/really, cannot/extreme(ly)] [11]. The three or five levels of the descriptive system are expressed with a five-digit code. For example, the Y-3L health state 11223 describes someone with no problems with *Mobility*, no problems with *Looking After Myself*, some problems with *Usual Activities*, some *Pain or Discomfort* and very *Worried, Sad or Unhappy*. The best health state described by the instrument is coded as 11111, describing ‘no problems’ in each of the dimensions [18]. Although the Y-3L has a preference-based score the Y-5L does not [[19], [20], [21], [22], [23]]. As such a level sum score (LSS) was used to describe the responses on the descriptive system where the level labels are treated as numeric data with the best possible score (1+1+1+1+1) =5 and the most severe score for the EQ-5D-Y-3L is (3+3+3+3+3) = 15. The other health states will have a LSS ranging between 5 and 15, with a larger score indicating a worse health state. Y-5L is similarly scored with a LSS ranging between 5 and 25 [24]. This is a crude measure with limitations [25,26] but gives some indication of the performance of the dimensions between the Y-3L and Y-5L. Given the differences between the Y-3L and adult (EQ-5D-3L) value sets, the LSS may give a better indication of performance than the adult EQ-5D-3L and EQ-5D-5L value sets [19,20].

#### 2.2.2 Pediatric Quality of Life Inventory (PedsQL)

The 23 item PedsQL Generic Core Scales for children aged 8-12 years and 13-18 years were used as appropriate [27]. Both age versions of the PedsQL consist of four dimensions of functioning: physical, emotional, social, and school with 8,5,5 and 5 items respectively. Each item is scored on a Likert scale from 0-4 (never a problem to almost always a problem). Items are reversed scored and transformed to a 0-100 scale: 0=100, 1=75, 2=50, 3=25, 4=0. Dimension scores are calculated by a sum of the item scores divided by the total number of items. A total score is similarly generated by summing the dimension scores over the total number of dimensions giving an overall HRQoL score. Scores for scales with more than 50% missing data are not computed. A higher PedsQL score indicates a better HRQoL. The PedsQL is a profile measure which has been utilised previously to explore the concurrent validity of the EQ-5D-Y [[28], [29], [30]] and has shown to be valid and feasible in children and adolescents with CP [31].

#### 2.2.3 The Gross Motor Functioning Classification System (GMFCS)

The GMFCS is used to classify the level of motor functioning in persons diagnosed with CP [32]. This classification system gives a description of the child’s current motor function [33]. The GMFCS was the first validated classification system for children with CP to be tested for reliability and stability, in 1997, and has since been adopted for international use [34]. The GMFCS consists of five levels and is scored according to the age and functional ability of the child [35]. Level one is scored for children with the least mobility and functional problems, who can walk without restrictions but struggle with advanced gross motor functioning. Level two is scored for children who walk without assistive devices but struggle walking outdoors and in the community. Level three indicates walking independently with a handheld assistive device but difficulty walking in the community. Level four indicates the use of an assistive mobility device with the need of physical or powered assistance for mobility in the community. Level five is scored for children who are severely limited in self-mobility even with the use of assistive devices and require physical or powered assistance in most settings [33].

#### 2.2.4 Procedure

Ethics approval was obtained from the University of Cape Town, Faculty of Health Sciences, Human Research Ethics Committee (HREC 154_2019). The study was carried out in accordance with the declaration of Helsinki involving human participants [36] and the recommended Covid precautions.

Due to the constraints of the Covid pandemic children/adolescents were recruited through information leaflets that were sent home to them and their parents. For those who were willing and provided consent and assent the instruments were self-completed by the child/adolescent. The parent was asked to complete the socio-demographic information for their child and the children/adolescents were asked to self-complete the EQ-5D-Y-5L, PedsQL and Y-3L in that order. The Y-5L was presented first as Janssen et al (2008) found in the presentation of the adult measures that if the three-level version was presented first the additional levels on the five level were not considered [37]. The two versions were further separated by the PedsQL to reduce bias. The accompanying information clearly stated that parents should not influence the completion of the instruments.

### 2.3 Data management and analysis

The sample size was sufficiently powered (90%) to detect a moderate difference in correlations between instruments with a significance level of 0.05.

### 2.4 General performance and feasibility

The EQ-5D-Y responses and descriptive data were summarised in terms of frequency of responses. The feasibility was assessed by comparing the number of missing values for two EQ-5D-Y measures. The ceiling of the EQ-5D-Y was defined as the proportion of children/adolescents scoring no problems in a dimension or across all five dimensions (11111). The floor effect is the proportion of children/adolescents scoring the most severe problems for a dimension or across all five dimensions (55555/33333). The absolute reduction in proportion scoring no problems or the most severe problems from the 3L to the 5L was calculated and due to the small number of respondents reporting 11111 and 55555/33333 a percentage reduction was also calculated as (ceiling_Y-3L_- ceiling_Y-5L_)/ceiling_Y-3L_.

### 2.5 Redistribution properties of the EQ-5D-Y-3L to the EQ-5D-Y-5L

Paired dimension responses on the Y-3L and Y-5L were assessed for inconsistency using criteria established in previous studies comparing the adult EQ-5D versions [37,38]. A response pair was considered inconsistent if the Y-5L response was at least two levels away from the Y-3L response. To note the youth version differed from the adult version in that level 3 on the EQ-5D-Y-3L is semantically equivalent to level 4 on the EQ-5D-Y-5L, and not level 5, thus the redistribution of level 3 (EQ-5D-Y-3L) was considered to redistribute to level 3, 4 or 5 on the EQ-5D-Y-5L One expected that a lot of problems on the EQ-5D-Y-3L (level 3 EQ-5D-Y-3L) would redistribute to some problems (level 3 EQ-5D-Y-5L) a lot of problems (level 4 EQ-5D-Y-5L) or cannot (level 5 EQ-5D-Y-5L) on the EQ-5D-Y-5L. Similarly some problems (level 2 EQ-5D-Y-3L) would redistribute to a little bit of problems (level 2 EQ-5D-Y-5L), some problems (level 3 EQ-5D-Y-5L) or a lot of problems (level 4 EQ-5D-Y-5L) and no problems on (level 1 EQ-5D-Y-3L) would redistribute to no problems (level 1 EQ-5D-Y-5L) or a little bit of problems (level 2 EQ-5D-Y-5L). The proportion of EQ-5D-Y-3L and EQ-5D-Y-5L dimension response pairs were calculated for comparison

### 2.6 Discriminatory power

The Shannon Index (H') and the Shannon Evenness Index (J') were used to evaluate the discriminatory power of the Y-3L and Y-5L dimensions in terms of absolute and relative informativity [37,39]. The Shannon H’ and J’ indices are defined as follows:

$$H^{\prime }=\sum_{i=1}^{L}p_{i}\log_{2}p_{i}\;\text{and}\;J^{\prime }=\frac{H^{\prime }}{H\prime_{\max}}$$

where H’ is the absolute amount of informativity, L is the number of dimensions levels and p_*i*_ is the proportion of observations in the in the *i*^th^ level where Y-3L has three levels and Y-5L has five levels. A higher H’ index reflects that the descriptive system has captured more information, the maximum H’index is 1.58 and 2.32 on the Y-3L and Y-5L respectively. The Shannon Evenness index (J’) reflects the spread of the responses across levels regardless of the number of levels included in the descriptive system.

### 2.7 Convergent validity

Convergent validity between Y-3L and Y-5L was evaluated by individual dimension response-pairs, using Kendall tau B and Gamma correlations statistics.

### 2.8 Criterion validity

The concurrent validity of the dimension scores of the Y-3L and Y-5L were compared to similar individual PedsQL items and sub-scale scores using Spearman correlations (r_s_). PedsQL summary and total scores were compared to EQ-5D-Y VAS and LSS scores with the Pearson Correlation co-efficient. Correlation coefficients were interpreted according to Cohen: 0.1–0.29 low association, 0.3–0.49 moderate association and ≥0.5 high association [40].

Predictive validity was tested for the dimensions and LSS of the Y-3L and Y-5L across GMFCS scores (level 1 versus level 2 and 3 versus level 4 and 5) and across age-groups (8-11 years and 12-15 years) with Spearman rank order coefficients (r_s_). Children/adolescents with GMFCS level 1 have independent mobility and gross motor skills and were not grouped. GMFCS level 2 and 3 were grouped together as they both may require some degree of assistance with mobility. GMFCS level 4 and 5 were grouped together as they both require physical assistance for mobility. The known-group validity across health condition was further assessed for the median LSS and VAS score across the GMFCS and age groups by Kruskal Wallis and Mann-Whitney U test. It was anticipated that those with higher GMFCS scores would report more problems on EQ-5D-Y dimensions of physical health (*Mobility, Looking After Myself and Usual Activities*) but no difference on dimensions of *Pain or Discomfort, Worried, Sad or Unhappy* and the VAS general health rating [7,9,41].

All data analyses were conducted using SPSS Windows 27.0 (IBM SPSS Inc., Chicago, IL, USA) and Statistica Windows Version 13.0 (TIBCO Software Inc., Palo Alto, CA, USA).

## 3 Results

Research packs were sent home with 161 children/adolescents eligible to participate attending the four schools for children with special education needs. A total of 51 packs were returned all of whom provided consent and assent. Reasons for refusal of consent/assent was not collected.

The median age of the children/adolescents across the age groups was 11.8 years (IQR 9.9, 13.35). Sex of participants was similarly distributed across the total group (51% male). There were more children classified with a GMFCS level 1 (41%) than those with level 2 or 3 (37%) or with level 4 or 5 (22%).

### 3.1 General instrument performance and feasibility

The Y-3L and Y-5L responses across condition groups and for the total sample are presented in Table 1. Across all dimensions the Y-5L and Y-3L had ≤2% missing responses. The missing responses on the Y-5L were from three respondents whereas those on the Y-3L were from two respondents.

**Table 1:** EQ-5D-Y-3L and EQ-5D-Y-5L responses

		Y-5L		Y-3L	
		n	%	n	%
Mobility	No	9	18%	16	31%
	A little bit	22	43%		
	Some	8	16%	23	45%
	A lot	2	4%	11	22%
	Cannot	9	18%		
LAM	No	24	47%	24	47%
	A little bit	14	27%		
	Some	4	8%	19	37%
	A lot	0	0%	7	14%
	Cannot	9	18%		
UA	No	23	45%	21	41%
	A little bit	10	20%		
	Some	11	22%	23	45%
	A lot	3	6%	6	12%
	Cannot	3	6%		
P/D	No	29	57%	29	57%
	A little bit	10	20%		
	Some	11	22%	20	39%
	A lot	0	0%	2	4%
	Extreme	0	0%		
WSU	Not	33	65%	33	65%
	A little bit	15	29%		
	Quite	1	2%	16	31%
	Really	1	2%	2	4%
	Extremely	0	0%		
LSS	Median (IQR)	9 (7,12)		8 (6,9)	
VAS	Median (IQR)	98 (89,100)			

N=51, Mob: Mobility; LAM: Looking After Myself; UA: Usual Activities; P/D: Pain or Discomfort; WSU: Worried, Sad or Unhappy

The ceiling effect for reporting no problems across all dimensions (11111) was low on the Y-3L and Y-5L and showed a relative reduction of 4% when moving to the expanded Y-5L (Table 2). There was reduction in the reporting of no problems when moving from the Y-3L to the Y-5L for the dimension of *Mobility* only with a 44% relative reduction. No respondents scored most severe problems across all five dimensions (33333/55555) but there was less reporting of most severe problems at dimension level for all dimensions, except *Looking After Myself*, when moving from the Y-3L to the Y-5L. The absolute reduction of reporting most severe problem between the Y-3L and Y-5L was 4-6%, the relative reduction was however much higher (100%) for dimensions on *Pain or Discomfort* and *Worried, Sad or Unhappy.* There was however an increase in report of most severe problems for *Looking After Myself* when moving from the Y-3L to Y-5L.

**Table 2:** Ceiling effect for the EQ-5D-Y-3L and EQ-5D-Y-5L

	Y-3L		Y-5L		absolute reduction	relative reduction
	n	%	n	%		
**Ceiling effect**						
11111	7	14%	6	12%	2%	14%
Mobility	16	31%	9	18%	14%	44%
LAM	24	47%	24	47%	0%	0%
UA	21	41%	23	45%	-4%	-10%
P/D	29	57%	29	57%	0%	0%
WSU	33	65%	33	65%	0%	0%
**Floor effect**						
33333/55555	0		0			
Mobility	11	22%	9	18%	4%	18%
LAM	7	14%	9	18%	-4%	-29%
UA	6	12%	3	6%	6%	50%
P/D	2	4%	0	0%	4%	100%
WSU	2	4%	0	0%	4%	100%

N=51, LAM: Looking After Myself; UA: Usual Activities; P/D: Pain or Discomfort; WSU: Worried, Sad or Unhappy

### 3.2 Redistribution properties of the EQ-5D-Y-3L to the EQ-5D-Y-5L

The dimension of *Usual Activities* had the highest and *Looking After Myself* the lowest number of inconsistent responses when moving from the Y-3L to the Y-5L (Table 3). Seventeen (33%) of the total inconsistent responses are due to moving from some problem on the Y-3L to no problem on the Y-5L, this accounts for 69% of all the inconsistent response pairs.

**Table 3:** Cross tabulation for the EQ-5D-Y-3L and EQ-5D-Y-5L dimension scores

Y-3L	Y-5L					Inconsistent responses	
**Mobility**	no	a little bit	some	a lot	cannot	n	%
no	6	8	***1***	***0***	***1***	5	10%
some	***3***	13	7	0	***0***		
a lot	***0***	***0***	0	2	8		
**LAM**	no	a little bit	some	a lot	cannot		
no	22	2	***0***	***0***	***0***	3	6%
some	***2***	12	4	0	***1***		
a lot	***0***	***0***	0	0	7		
**UA**	no	a little bit	some	a lot	cannot		
no	17	2	***2***	***0***	***0***	7	14%
some	***4***	8	8	1	***1***		
a lot	***1***	***0***	1	2	2		
**P/D**	no	a little bit	some	a lot	extreme		
no	25	3	***0***	***0***	***0***	6	12%
some	***4***	5	11	0	***0***		
a lot	***0***	***2***	0	0	0		
**WSU**	not	a little bit	quite	really	extremely		
not	28	5	***0***	***0***	***0***	6	12%
a bit	***4***	9	1	1	***0***		
very	***1***	***1***	0	0	0		

n=51, bold and italicised indicates inconsistent responses.

LAM: Looking After Myself; UA: Usual Activities; P/D: Pain or Discomfort; WSU: Worried, Sad or Unhappy

### 3.3 Discriminatory power

Informativity of dimensions improves across all dimensions on the Y-5L compared to the Y-3L with an average improvement of 0.27 with similar evenness (Table 4). The discrimination on the dimension of *Worried, Sad or Unhappy* was low on both the Y-5L and Y-3L with very few respondents reporting severe problems as can be seen by the low J’ index. *Usual Activities* showed the greatest difference in spread of information between the Y-3L and Y-5L with similar levels for *Mobility, Looking After Myself* and *Pain or Discomfort.*

**Table 4:** Shannon Index (H’) and Shannon Evenness Index (J’) for the EQ-5D-Y-3L and EQ-5D-Y-5L dimensions.

	Y-5L		Y-3L			
	Shannon Index H'	Shannon Evenness Index J'	Shannon Index H'	Shannon Evenness Index J'	Difference in H'	Difference in J'
Mobility	−1.39	0.23	−1.05	0.28	−0.34	−0.05
LAM	−1.28	0.29	−1.00	0.36	−0.28	−0.07
UA	−1.34	0.31	−0.98	0.35	−0.36	−0.04
P/D	−1.10	0.34	−0.82	0.38	−0.28	−0.04
WSU	−0.87	0.22	−0.77	0.19	−0.10	0.03
Average difference					−0.27	−0.04

n=51 LAM: Looking After Myself; UA: Usual Activities; P/D: Pain or Discomfort; WSU: Worried, Sad or Unhappy

### 3.4 Convergent validity

Convergent validity between the paired Y-3L-Y-5L responses for each dimension was estimated with Gamma correlation coefficients ranging from 0.79 (*Mobility)* to 0.99 (*Looking After Myself*). Kendall tau B coefficients similarly showed high correlations ranging from 0.53 (*Pain or Discomfort)* to 0.85 (*Looking After Myself)* (Table 5).

**Table 5:**
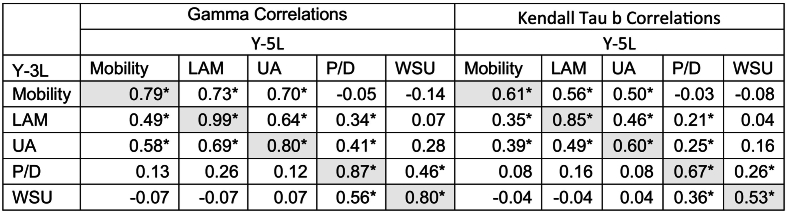
Convergent validity of the Y-3L and Y-5L dimension scores

n=51. *p<0.05. LAM: Looking After Myself; UA: Usual Activities; P/D: Pain or Discomfort; WSU: Worried, Sad or Unhappy, VAS: Visual Analogue Scale.

### 3.5 Criterion validity

There were missing responses from ten respondents on the PedsQL scale, four of which did not complete any of the PedsQL items and were excluded from analysis. The missing item responses ranged from 9-13% with the items of household chores, low energy levels, afraid or scared, worry and trouble keeping up with schoolwork having the highest number of missing responses. Table 6 shows that the Y-3L and Y-5L had similar moderate association with similar items on the PedsQL. Table 7 shows the concurrent validity of the Y-3L and Y-5L VAS and LSS with the PedsQL scores. The Y-3L and Y-5L LSS and the PedsQL total score and physical summary scores had significant moderate to high associations for children/adolescents with CP. The psychosocial summary score nor either of its sub-scores were significantly associated with Y-3L or Y-5L LSS except the school sub-scores which showed a low correlation with the Y-3L LSS. The VAS score did not show any association with the PedsQL.

**Table 6:**
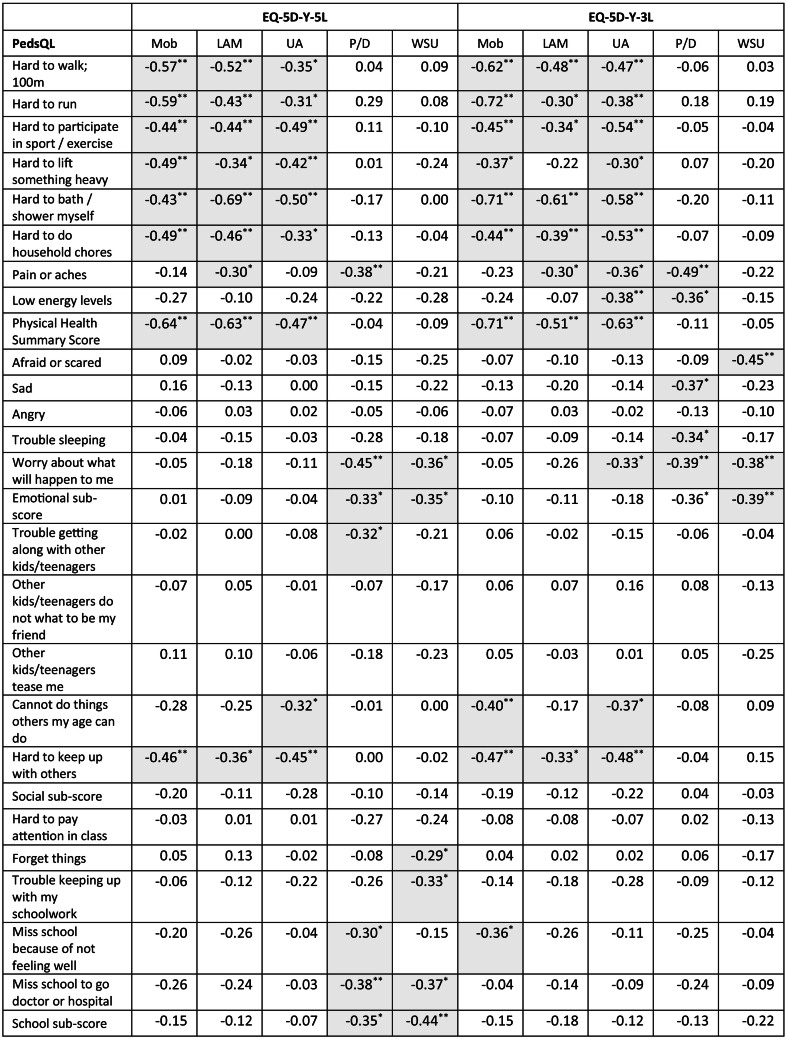
Spearman Correlation of EQ-5D-Y-5L and EQ-5D-Y-3L dimension scores and PedsQL item and sub-scale scores

n=51, * p<0.05 and **p<0.001 (2-tailed). Cells shaded in grey have a moderate association >0.30, correlations are negative as a higher PedsQL score indicates a better HRQoL. 1 PedsQL score not computed as >50% missing data and 4 respondents did not complete the PedsQL. Mob: Mobility; LAM: Looking After Myself; UA: Usual Activities; P/D: Pain or Discomfort; WSU: Worried, Sad or Unhappy, VAS: Visual Analogue Scale.

**Table 7:**
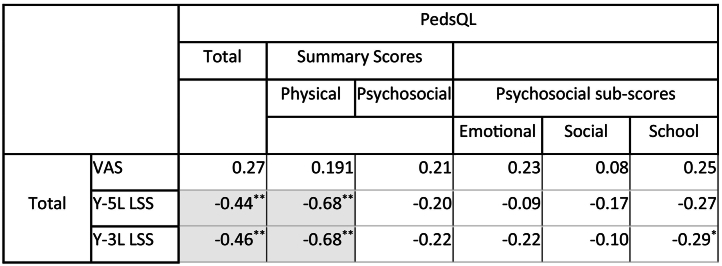
Summary of concurrent validity with the PedsQL summary and sub-scores, EQ-5D-Y VAS and LSS scores with Pearson correlation.

N=51, *p<0.05 and **p< 0.001 level (2-tailed). Cells shaded in grey have a moderate association >0.30, VAS: Visual Analogue Scale; LSS: Level Sum Score; GP: General Population.

The hypotheses for predictive validity or known-group validity were confirmed and presented in Table 8. There were no differences in GMFC level across age groups (p=0.804). When considering GMFCS scores there were no significant differences between GMFCS and *Pain or Discomfort* and *Worried, Sad or Unhappy*. Physical dimensions of *Mobility, Looking After Myself* and *Usual Activities* were significantly associated with GMFCS with more problems reported across these dimensions with a higher GMFCS score, indicating less independent mobility. Furthermore, the Y-5L LSS was significantly different for those with a GMFCS level 4 or 5 compared to those with a level 1 (p<0.001) and level 2 and 3 (p<0.001). Similarly the Y-3L LSS was significantly different for those with GMFCS level 4 or 5 compared to those with level 1 (p=0.009) and level 2 and 3 (p=0.036). There were no significant differences between GMFCS level when rating general health on the VAS (p=0.092). Both the Y-5L LSS (p<0.001) and Y-3L LSS (p=0.009) were able to discriminate between the least severe GMFCS score (1) and the most severe (level 4 and 5) and between children classified with GMFCS level 2 and 3 and those classified as a level 4 or 5 (Y-5L p<0.001 and Y-3L p=0.036). Neither measure were able to differentiate between the less severe levels of GMFCS level 1 or level 2 and 3.

**Table 8:** Spearman rank correlation of EQ-5D-Y-3L and EQ-5D-Y-5L dimension scores and GMFCS scores

Variable	Y-5L	Y-3L
	GMFCS	GMFCS
Mobility	0.58*	0.53*
LAM	0.45*	0.33*
UA	0.31*	0.39*
P/D	−0.15	−0.09
WSU	−0.13	−0.24

N=51, *p<0.05 (2-tailed), GMFCS: Gross Motor Classification System; LAM: Looking After Myself; UA: Usual Activities; P/D: Pain or Discomfort; WSU: Worried, Sad or Unhappy

## 4 Discussion

The aim of this study was to investigate the feasibility, redistribution and discriminatory power of dimension responses, convergent validity, and criterion validity (concurrent and predictive validity) of Y-3L and the Y-5L in children/adolescents with CP.

The Y-5L and Y-3L are both feasible instruments for measuring health status in children/adolescents with CP with a low percentage of missing responses on self-complete. At an item level the Y-5L and Y-3L had 2% or less missing responses similar to previous report in the Y-3L [9]. This was considerably less than the PedsQL with 9-13% missing responses across items. This was further evident from the number of respondents who had missing responses with only three (6%) on the Y-5L, two (4%) on the Y-3L and ten (20%) on the PedsQL.

As expected, in respondents with a health condition, the ceiling effect with reporting no problems on all dimensions (11111) was low on the Y-3L and Y-5L [9] and the reduction from the Y-3L to the Y-5L was similar to that found by Wong et al (2019) in a group of children with idiopathic scoliosis [15] and on the Y-3L from children/adolescents with CP [9,41]. At dimension level the reporting of no problems was greatly reduced for *Mobility* on the Y-5L compared to the Y-3L*.* There was similarly a high relative reduction in reporting of most severe problems in dimensions of *Mobility, Usual Activities, Pain or Discomfort* and *Worried, Sad or Unhappy.* The reduction in ceiling and floor effects on the Y-5L has important implications for its use as a health status measure for population health but also for detecting change in clinical trials or as a routine outcome measure [7] and it is recommended that the Y-5L is further tested for reliability and responsiveness.

Although there were respondents who reported more severe problems on the Y-5L than on the Y-3L for *Looking after Myself* this could be attributed to the change of the most severe levels from ‘a lot’ to ‘cannot’ which was similarly reported in children/adolescents receiving orthopaedic management [14]. When considering the paired Y-5L – Y-3L scores *Looking After Myself* did have the highest convergent validity and as this was not observed across dimensions it is likely not due to the order effect. The reduction in ceiling effect and inconsistency of responses both show that there were many respondents (33%) who moved from some problems on the Y-3L to no problems on the Y-5L across all dimensions. This could be attributed to the order effect with the Y-5L being completed first and the Y-3L completed last and after the PedsQL instrument. The inconsistencies across dimensions (6%-12%) was higher than those with idiopathic scoliosis (0-4%) [15] but lower than those receiving acute orthopaedic management (11-27%) [14]. Despite these inconsistencies with redistribution of the Y-3L and Y-5L responses the paired dimension scores showed a high convergent validity across all dimensions.

The discriminatory power of the Y-5L showed a large improvement with the expanded levels of the Y-5L compared to the Y-3L (average H’=0.27) and was similar to the finding in respondents receiving acute orthopaedic intervention (average H’=0.267) [14] and larger than those with idiopathic scoliosis (average H’=0.024) [15]. The evenness of the distribution of responses on the Y-5L (average J’=0.045) was retained. Looking at individual dimensions the distribution of scores was however particularly poor on the dimension of *Worried, Sad or Unhappy* across the Y-5L and Y-3L as few respondents reported severe problems. This is in keeping with results from the Y-3L [9,41] and a systematic review suggesting that mental health disorders are less prevalent in children with CP who do not have intellectual disability and are within a supportive environment [42].

To our knowledge, this was the first study to compare the EQ-5D-Y instruments to the PedsQL generic measure in children with CP. The Y-3L and Y-5L dimensions both showed similar concurrent validity with moderate correlations with PedsQL items. The Y-3L and Y-5L LSS showed moderate corelation with the PedsQL total score (0.46 and 0.44 respectively) which is similar to the average correlation of 0.45 found when between PROMs in a meta-analysis [7] but better than the poor correlation reported between the CHU-9D and EQ-5D-Y-3L utility scores [9]. The criterion validity of the Y-5L and Y-3L is further supported with differentiation between dimensions of physical health (*Mobility, Looking After Myself* and *Usual Activities)* and the LSS with the GMFCS score which was similarly found on the HUI-2 and HUI-3 [7] and on the Y-3L but not on the CHU-9D [9]. The correlation coefficients with the GMFCS are higher for the Y-5L dimensions scores than the Y-3L further supporting that it would be the more preferred instrument.

Due to the limitations of the Covid pandemic on recruitment of children/adolescents with CP at the schools there may be non-response bias. Although parents and children/adolescents were explicitly instructed to complete the measures on their own without influence from others there was no way to ensure this. The relatively small sample of children with CP in this study limited more extensive analysis and it is recommended that future studies include a larger group of children with consideration for spread across the GMFCS levels. The LSS used is a crude summary of dimensions and indicates the performance of the instruments in the absence of utility values for the Y-5L with known limitations [28,29]. The development of a utility score or mapping of scores from the Y-3L to the Y-5L would be of great benefit for direct comparison of these two instruments.

## 5 Conclusion

The results of this research support the use of patient reported outcomes, particularly the EQ-5D-Y-5L, for children and adolescents with CP. The Y-5L showed a notable reduction in ceiling and floor effects, improved discriminatory power, higher criterion validity with the GMFCS and similar concurrent validity with the PedsQL as the Y-3L. It is recommended that the Y-5L is further tested for reliability and responsiveness in this population group so that its utility for detecting change in clinical trials or as a routine outcome measure can be determined.

## Consent for publication

No identifying information has been included in this manuscript. All participants consented to the publication of the analysed data.

## Availability of data and materials

The datasets used and/or analysed during the current study are available from the corresponding author on reasonable request.

## Funding

10.13039/501100006419EuroQol Research Foundation project EQ20180730

## Authors' contributions

JV and DS both contributed towards the proposal development, conception and design of the study, ethical submission, data collection, data analysis and final write up.

## Declaration of Competing Interest

JV and DS are members of the EuroQoL Research Foundation. This did not influence the reporting of the research study. The views expressed by the authors in the publication do not necessarily reflect the views of the EuroQol Group.
